# Angiomotin-Like 1 Links Paramyxovirus M Proteins to NEDD4 Family Ubiquitin Ligases

**DOI:** 10.3390/v11020128

**Published:** 2019-01-31

**Authors:** Greeshma Ray, Phuong Tieu Schmitt, Anthony P. Schmitt

**Affiliations:** 1 Department of Veterinary and Biomedical Sciences, The Pennsylvania State University, University Park, PA 16802, USA; greeshma.ray@gmail.com (G.R.); pts13@psu.edu (P.T.S.); 2 Center for Molecular Immunology and Infectious Disease, The Pennsylvania State University, University Park, PA 16802, USA

**Keywords:** paramyxovirus, assembly, budding, late domain, PPXY, angiomotin, AMOTL1, NEDD4

## Abstract

To define the links between paramyxovirus budding and cellular ESCRT machinery, we previously identified angiomotin-like 1 (AMOTL1) in a screen for host factors that bind to the matrix (M) protein of parainfluenza virus 5 (PIV5). This protein harbors three L/PPXY sequences, allowing it to interact with WW domain containing proteins including NEDD4 family members. We hypothesize that paramyxoviruses use AMOTL1 as a linker to indirectly recruit the same NEDD4 ubiquitin ligases for budding that other enveloped viruses recruit directly through their PPXY late domains. In support of this hypothesis, we found that AMOTL1 could link together M proteins and NEDD4 family proteins in three-way co-IP experiments. Both PIV5 and mumps virus M proteins could be linked to the NEDD4 family proteins NEDD4-1, NEDD4L, and NEDL1, provided that AMOTL1 was co-expressed as a bridging protein. AMOT and AMOTL2 could not substitute for AMOTL1, as they lacked the ability to bind with paramyxovirus M proteins. Attachment of a PPXY late domain sequence to PIV5 M protein obviated the need for AMOTL1 as a linker between M and NEDD4 proteins. Together, these results suggest a novel host factor recruitment strategy for paramyxoviruses to achieve particle release.

## 1. Introduction

Paramyxoviruses form a diverse group of enveloped negative-strand RNA (nsRNA) viruses that are among the most common causes of acute respiratory tract infections in people worldwide [1]. These viruses spread infections primarily via particles that bud from the plasma membranes of infected cells. Particle budding is coordinated by viral matrix (M) proteins, which self-assemble to form layers at discrete assembly sites underlying infected cell plasma membranes and recruit other viral components to these locations, including the viral glycoproteins, and viral ribonucleoprotein (RNP) complexes [2,3]. 

Many enveloped viruses do not encode the machinery that is needed to bud particles. Instead, host machinery from the Endosomal Sorting Complex Required for Transport (ESCRT) pathway is recruited to allow efficient virus exit [4,5]. Three types of late domains, all originally identified within the Gag proteins of retroviruses, can serve to mediate binding with ESCRT components. P(T/S)AP directs binding to the ESCRT-I protein TSG101, and YPXL directs binding to the ESCRT-bridging protein ALIX [4,6]. The third type of retroviral late domain has the sequence PPXY, and this directs binding to WW domain-containing E3 ubiquitin ligases from the NEDD4 family [4,6,7]. Regardless of the type or combination of late domains employed, the ultimate consequence is a cascade of protein-protein interactions that eventually recruit ESCRT-III, which forms polymeric membrane-bound filaments that are thought to drive constriction of the bud neck leading to membrane fission [4,8]. ESCRT complexes are dismantled and recycled with the aid of VPS4 AAA-ATPases, and disruption of ESCRT function using dominant-negative VPS4 protein mutants blocks the budding of many retroviruses [6].

Matrix proteins of many negative-strand RNA viruses contain the same late domain sequences that are utilized by retroviruses, suggesting that the overall strategy of ESCRT recruitment for budding is well-conserved. Matrix proteins of VSV [9,10,11], Ebola virus [12,13,14], lymphocytic choriomeningitis virus [15], and Lassa fever virus [15] all contain PPXY sequences together with P(T/S)AP sequences, while rabies virus M protein contains PPXY without P(T/S)AP [16]. Paramyxoviruses, however, generally lack PPXY, P(T/S)AP, and YPXL sequences within their M proteins. Nonetheless, our group has demonstrated ESCRT-dependent budding for the paramyxoviruses mumps virus [17] and PIV5 [17,18], and others have demonstrated ESCRT-dependent budding for Newcastle disease virus (NDV) [19], Sendai virus [20,21], human parainfluenza virus 1 (HPIV1) [22], and Nipah virus [23]. 

**Figure 1:**
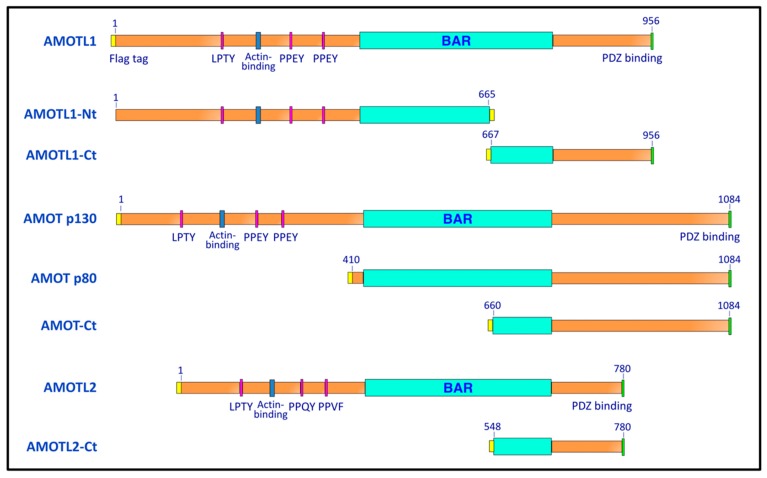
Schematic illustration of the angiomotins and angiomotin-derived polypeptides. Numbers indicate the span of amino acid residues that are contained within each construct. All constructs are Flag-tagged, with the positions of the tags indicated by the yellow regions. AMOT p80 is a naturally-occurring splice variant of AMOT that lacks the entire PPXY-containing N-terminal region, but retains the consensus BAR and PDZ-binding domains.

We previously identified angiomotin-like 1 (AMOTL1) as a PIV5 M-interacting host factor using yeast two-hybrid screens, as part of an effort to define links between paramyxovirus budding and the ESCRT pathway [24]. AMOTL1 belongs to a family of angiomotin proteins, together with angiomotin (AMOT) and angiomotin-like 2 (AMOTL2) [25] (Figure 1). All three angiomotins have well-characterized functions in the regulation of endothelial cell migration and polarity [25,26,27]. In epithelial cells, all three members associate with tight junctions through interaction via their PDZ binding domains with the tight junction-associated proteins PATJ, PALS1, and RICH1, and all three are found localized to junctional complexes as well as apical membranes [25,28,29,30]. All three angiomotin proteins contain PPXY motifs in their N-terminal regions [25] (Figure 1). AMOT harbors two canonical PPEY sequences, plus a recently-identified, unconventional LPTY sequence [31], and this arrangement is conserved between AMOT and AMOTL1. AMOTL2 contains PPQY and the slightly divergent PPVF, in addition to LPTY [25]. Since AMOTL1 has the ability to bind with viral M protein on the one hand, and on the other hand bind to WW domain-containing proteins via PPXY, it is plausible that paramyxoviruses may use AMOTL1 as a linker to indirectly recruit the same WW domain-containing NEDD4 ubiquitin ligases for budding that other enveloped viruses recruit directly through their PPXY late domains.

Such an arrangement would be analogous to that recently-described by Wes Sundquist and colleagues for the budding of HIV-1. There, AMOT was identified as a bridge between HIV-1 Gag and the WW domain-containing NEDD4 ubiquitin ligase NEDD4L [32]. NEDD4L expression was able to rescue the budding of PTAP-defective Gag budding despite the absence of PPXY within Gag [33,34]. Rescue of PTAP-defective Gag budding by NEDD4L was enhanced when AMOT was overexpressed and diminished when AMOT expression was knocked down [32]. While TSG101 depletion arrested HIV-1 budding at a late, “lollipop” stage of budding as determined by TEM, AMOT depletion arrested budding at an earlier “half-moon” stage consistent with incomplete wrapping of the membrane around the Gag shell [32]. This suggests a model for HIV-1 budding in which there is stepwise recruitment of different host factors with AMOT recruitment, NEDD4L binding, and perhaps Gag ubiquitination occurring at the earlier stages, leading to subsequent binding by TSG101, indirect recruitment of ESCRT-III, and membrane fission [32].

Here, we have explored the potential of angiomotins to indirectly link NEDD4 family proteins to paramyxovirus M proteins. In contrast to HIV-1 Gag protein, which was found capable of interacting with any of the three angiomotins, we show here that PIV5 and mumps virus M proteins bind selectively to AMOTL1 and cannot bind to either AMOT or AMOTL2. Multiple NEDD4 family proteins were found to be capable of binding to AMOTL1, and expression of AMOTL1 allowed indirect recruitment of these NEDD4 proteins to the viral M proteins. These results provide the first demonstration of indirect physical recruitment of NEDD4 ubiquitin ligases to paramyxovirus M proteins.

## 2. Materials and Methods

### 2.1. Plasmids

The plasmids pCAGGS-PIV5 M, pCAGGS-PIV5 NP, and pCAGGS-PIV5 HN have been described before [35], as have plasmids pCAGGS-MuV M, pCAGGS-MuV NP, and pCAGGS-MuV F [17]. pCAGGS-PIV5 M-PPXY was generated by PCR modification of pCAGGS-PIV5 M to append the Rous sarcoma virus late domain (amino acid sequence TASAPPPPYVG) to the C-terminal end of M.

Plasmids pCAGGS-AMOTL1, pCAGGS-AMOTL1-Ct, and pCAGGS-AMOTL1-Nt have been described previously [24]. cDNAs corresponding to human AMOT p130 (NCBI NM_001113490), AMOT p80 (NCBI NM_133265), and AMOTL2 (NCBI NM_016201) were kind gifts from Dr. Wesley Sundquist and were obtained via the DNASU repository (dnasu.org). These were modified by PCR to append Flag (DYKDDDDK) tags to their N-termini and subcloned into the pCAGGS vector [36] to generate pCAGGS-AMOT p130, pCAGGS-AMOT p80, and pCAGGS-AMOTL2. AMOT p130 cDNA was further modified by PCR to generate AMOT-Ct having boundaries as indicated in Figure 1. AMOTL2 cDNA was likewise modified to generate AMOTL2-Ct. Boundary points for AMOT-Ct and AMOTL2-Ct were selected based on alignments of the amino acid sequences to that of AMOTL1-Ct using ClustalW2 [37]. pCAGGS-AMOTL1-AAEY_310_ was generated by PCR mutagenesis of pCAGGS-AMOTL1, changing the PPEY sequence starting at amino acid residue 310 to AAEY. Likewise, pCAGGS-AMOTL1-AAEY_367_ was generated, changing the PPEY sequence starting at residue 367 to AAEY. pCAGGS-AMOTL1-AAEY_310,367_ changes both of these PPEY sequences to AAEY. This double mutant was further modified by PCR to generate the triple mutant pCAGGS-AMOTL1 ∆L/PPXY, in which the LPTY sequence starting at residue 188 is changed to AATY and the PPEY sequences starting at residues 310 and 367 are both changed to AAEY. DNA sequencing of the entire genes was carried out to verify their identities (Eurofins Genomics, Louisville, KY, USA).

cDNAs corresponding to NEDD4L (isoform 2, NCBI NP_001138436), as well as the catalytically inactivated NEDD4L^CA^ harboring the C942A mutation, were obtained from Dr. Wesley Sundquist via the DNASU repository. cDNAs corresponding to NEDD4-1 (NCBI BC152452) and NEDL1 (NCBI BC151227) were purchased from Transomic Technologies (Huntsville, AL, USA). Each of these was modified by PCR to add a Myc epitope tag to the N-terminus, and subcloned into pCAGGS vector.

### 2.2. Antibodies

Monoclonal antibodies M-f, HN1b, and NPa, specific to the PIV5 M, HN, and NP proteins, respectively, have been described before [38] and were kind gifts of Dr. Richard Randall, St. Andrews University, St. Andrews, Scotland, UK. Mumps virus M and F proteins were detected using polyclonal anti-peptide antibodies that have been described before [17]. Monoclonal antibody NP(19a), specific to the mumps virus NP protein, was a kind gift of Dr. Biao He, University of Georgia. Polyclonal anti-AMOTL1, raised against full-length *Escherichia coli*-expressed human AMOTL1 protein, has been described before [24]. Monoclonal antibody specific to the Myc tag was purchased from Life Technologies (ThermoFisher Scientific, Waltham, MA, USA), monoclonal antibody specific to the Flag tag was purchased from Origene, Rockville, MD, USA, and anti-flag M2 magnetic beads were purchased from Sigma-Aldrich (St. Louis, MO, USA).

### 2.3. Co-Immunoprecipitation

293T cells grown in 10-cm-diameter dishes to 70–80% confluency in Dulbecco’s modified Eagle medium (DMEM) supplemented with 10% fetal bovine serum (FBS), were transfected with pCAGGS plasmids encoding PIV5 or mumps virus M proteins (0.8 µg/dish), pCAGGS plasmids encoding AMOT, AMOTL1, AMOTL2, or derivatives (1.0 µg/dish), and/or pCAGGS plasmids encoding NEDD4-1, NEDD4L^CA^, or NEDL1 (1.5 µg/dish). Cells were transfected using Lipofectamine-Plus reagents (Life Technologies) per manufacturer’s protocol. At 24 h post-transfection, cells were starved for 20 min in DMEM containing 2% FBS and 1/10 the normal amount of methionine and cysteine followed by labeling for 3 h in the same medium supplemented with 37 µCi of [^35^S] Promix/mL (Perkin Elmer, Waltham, MA, USA). Cells were harvested and mixed with lysis buffer (20 mM Tris-HCl, pH 7.5, 150 mM NaCl, 1 mM EDTA, 0.5% NP-40, 5% glycerol, 1 mM PMSF). The resulting cell lysates were clarified by centrifugation, followed by rocking for 3–4 h at 4 °C in the presence of anti-myc monoclonal antibody for pulldown of NEDD4 family proteins or anti-Flag M2 magnetic beads for pulldown of angiomotins. Immune complexes were collected by centrifugation after incubation with protein A sepharose beads for 0.5 h, and washed 3 times with lysis buffer. Proteins were separated by SDS-PAGE using 10% gels, and were detected using a Fuji FLA-7000 phosphorimager (FujiFilm Medical Systems, Stamford, CT, USA).

Alternatively, for co-immunoprecipitation followed by immunoblot detection, 293T cells were transfected as described above. Cells were harvested at 24 h post-transfection, followed by cell lysis, incubation with antibodies, collection of immune complexes using protein A sepharose, and SDS-PAGE as described above. Proteins were transferred to PVDF membranes and immunodetection was carried out using the following antibody reagents: rabbit polyclonal antibody specific to AMOTL1 for detection of AMOTL1 and derivatives; anti-Flag tag antibody for detection of AMOT, AMOTL2, and derivatives; anti-Myc tag antibody for detection of NEDD4-1, NEDD4L^CA^, and NEDL1; monoclonal antibodies M-f and NPa for detection of PIV5 M and NP proteins; monoclonal antibody NP(19a) for detection of mumps virus NP protein; and rabbit polyclonal anti-peptide antibodies for detection of mumps virus M and F proteins. Blots were washed, incubated with alkaline phosphatase-conjugated goat anti-rabbit or goat anti-mouse secondary antibodies (Jackson ImmunoResearch Laboratories, Inc., West Grove, PA, USA), and imaged using a Fuji FLA-7000 laser scanner.

### 2.4. Measurements of VLP Production

To generate PIV5 VLPs, 293T cells grown in 6-cm dishes to 70–80% confluency in DMEM supplemented with 10% fetal bovine serum were transfected with pCAGGS plasmids encoding PIV5 M protein or M-PPXY protein (0.4 µg/dish), PIV5 NP protein (0.1 µg/dish), and PIV5 HN protein (1.5 µg/dish). Mumps VLPs were generated using pCAGGS plasmids encoding mumps virus M protein (0.4 µg/dish), mumps virus NP protein (0.1 µg/dish), and mumps virus F protein (0.1 µg/dish). In some cases, the viral components were transfected together with additional pCAGGS plasmids encoding AMOT-Ct, AMOTL1-Ct, or AMOTL2-Ct (all at 0.1 µg/dish). Transfections were carried out in Opti-MEM using Lipofectamine-Plus reagents. At 24 h p.t., the culture medium was replaced with DMEM containing 2% FBS, 1/10 the normal amount of methionine and cysteine, and 37 µCi of [^35^S] Promix/mL. After an additional 16–18 h, cell and media fractions were collected. VLPs from the culture media fractions were pelleted through 20% sucrose cushions, resuspended, floated to the tops of sucrose flotation gradients, pelleted again, and then resuspended in SDS-PAGE loading buffer containing 2.5% (*w*/*v*) dithiothreitol, as described previously [39]. Cell lysate preparation and immunoprecipitation of proteins from the cell lysate fractions were carried out as described [35]. Antibodies for immunoprecipitation of viral proteins are the same as those described above for co-immunoprecipitation. Anti-Flag M2 magnetic beads were used to immunoprecipitate angiomotin-derived polypeptides. The precipitated proteins and VLPs were separated on 10% SDS gels and detected using a Fuji FLA-7000 phosphorimager. VLP production efficiency was calculated as the quantity of M protein in purified VLPs divided by the quantity of M protein in the corresponding cell lysate fraction, normalized to the value obtained in the positive control experiment.

Alternatively, for VLP production followed by immunoblot detection, 293T cells were transfected as described above. Cell and media fractions were harvested at 40 h post-transfection. VLPs in media fractions were purified by centrifugation through sucrose cushions and flotation on sucrose gradients as described above. Cell lysates and purified VLPs were fractionated on 10% SDS gels, and proteins were transferred to PVDF membranes for immunodetection using the same primary and secondary antibody reagents described above for co-immunoprecipitation. Blots were imaged using a Fuji FLA-7000 laser scanner.

## 3. Results

### 3.1. Paramyxovirus M Proteins Bind Selectively to AMOTL1

**Figure 2:**
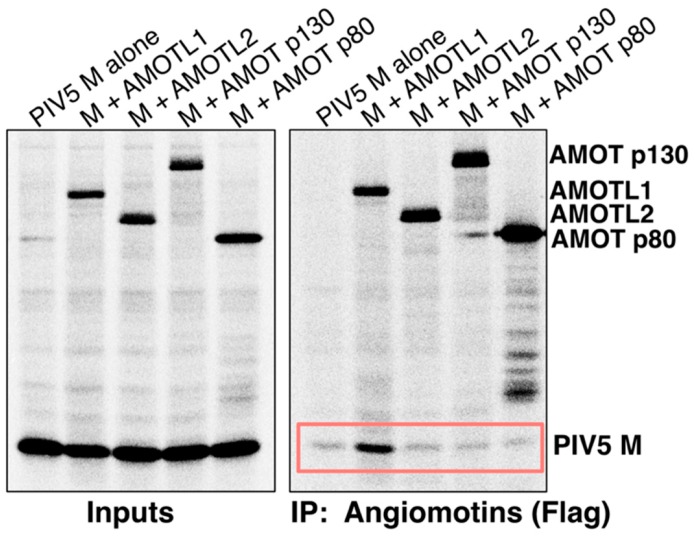
PIV5 M protein binds to AMOTL1, but not to AMOT or AMOTL2. 293T cells were transfected to produce PIV5 M protein together with the indicated Flag-tagged angiomotins. Proteins synthesized in the transfected cells were metabolically labeled with ^35^S, and cells were lysed in a solution containing 0.5% NP-40. Immunoprecipitation was carried out using a combination of anti-Flag and anti-PIV5 M antibodies (left), or using Flag antibody only (right). Proteins were fractionated on SDS gels and detected using a phosphorimager. Boxed region denotes M co-precipitation.

We compared the abilities of AMOT, AMOTL1, and AMOTL2 to bind paramyxovirus M proteins. 293T cells were transfected to produce Flag-tagged angiomotins (illustrated in Figure 1), together with PIV5 M protein. Angiomotins were immunoprecipitated using Flag resin, and co-precipitation of M protein was evaluated (Figure 2). Clear M protein co-precipitation was observed with AMOTL1, but no M co-precipitation was observed with AMOT. Both full-length AMOT (p130) and its smaller isoform (p80) were tested, and neither interacted with PIV5 M. AMOTL2 was also evaluated, and no interaction with PIV5 M was detected (Figure 2). Hence, PIV5 M protein binding to angiomotins was selective to AMOTL1.

**Figure 3:**
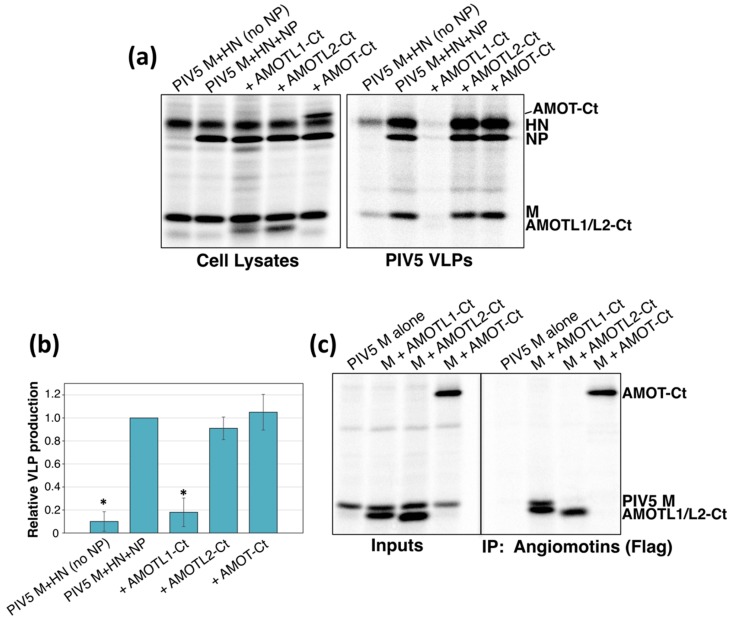
M-binding, AMOTL1-derived polypeptides inhibit the budding of PIV5 VLPs, but the corresponding AMOT- and AMOTL2-derived polypeptides do not. (**a**) 293T cells were transfected to produce PIV5 M, HN, and NP proteins together with the indicated angiomotin-derived polypeptides. After metabolic labeling of cells, lysates were prepared and viral proteins and angiomotin-derived polypeptides were immunoprecipitated. VLPs from culture supernatants were purified by centrifugation through sucrose cushions followed by flotation on sucrose gradients. Purified VLPs were loaded directly onto SDS gels without immunoprecipitation, and proteins were visualized using a phosphorimager. (**b**) Four independent experiments were performed as described for panel (**a**), and VLP production efficiencies were calculated as the amount of viral M protein detected in VLPs divided by the amount of M protein detected in the corresponding cell lysate fraction and were normalized to the values obtained in the absence of any polypeptide co-expression. Error bars indicate standard deviations. Differences from the values obtained in the absence of polypeptide co-expression were assessed for statistical significance by using a two-tailed Student’s *t*-test, and *p* values less than 0.01 are denoted by an asterisk. (**c**) 293T cells were transfected to produce PIV5 M protein together with the indicated Flag-tagged angiomotin-derived polypeptides, and co-immunoprecipitation was evaluated as described in the legend to Figure 1.

Previous studies have mapped the M binding activity of AMOTL1 to its C-terminal region. For example, AMOTL1-Ct (illustrated in Figure 1), corresponding to the C-terminal 30% of AMOTL1, readily binds to PIV5 M protein [24]. When expressed together with viral proteins, AMOTL1-Ct acts as a potent inhibitor of PIV5 VLP budding [24], perhaps by competing with full-length endogenous AMOTL1 for binding with M. Here, we tested if the corresponding C-terminal fragments derived from AMOT or AMOTL2 would affect PIV5 VLP budding. These polypeptides were expressed in 293T cells together with PIV5 M, NP, and HN proteins. This combination of viral proteins leads to very efficient VLP production [35] (Figure 3a). Omission of the viral NP protein substantially reduces VLP production, and this served as a reference point for poor budding in these experiments (Figure 3, first lane). Expression of AMOTL1-Ct substantially reduced VLP production, such that the impact on VLP levels was similar to that observed in the absence of NP (Figure 3a). However, expression of AMOT-Ct or AMOTL2-Ct had no substantial effect on VLP production (Figure 3a). Co-immunoprecipitation confirmed that AMOT-Ct and AMOTL2-Ct failed to interact with PIV5 M, consistent with their full-length counterparts (Figure 3b). Hence, the ability of angiomotin-derived polypeptides to inhibit VLP budding correlated with their ability to bind to the viral M protein.

**Figure 4:**
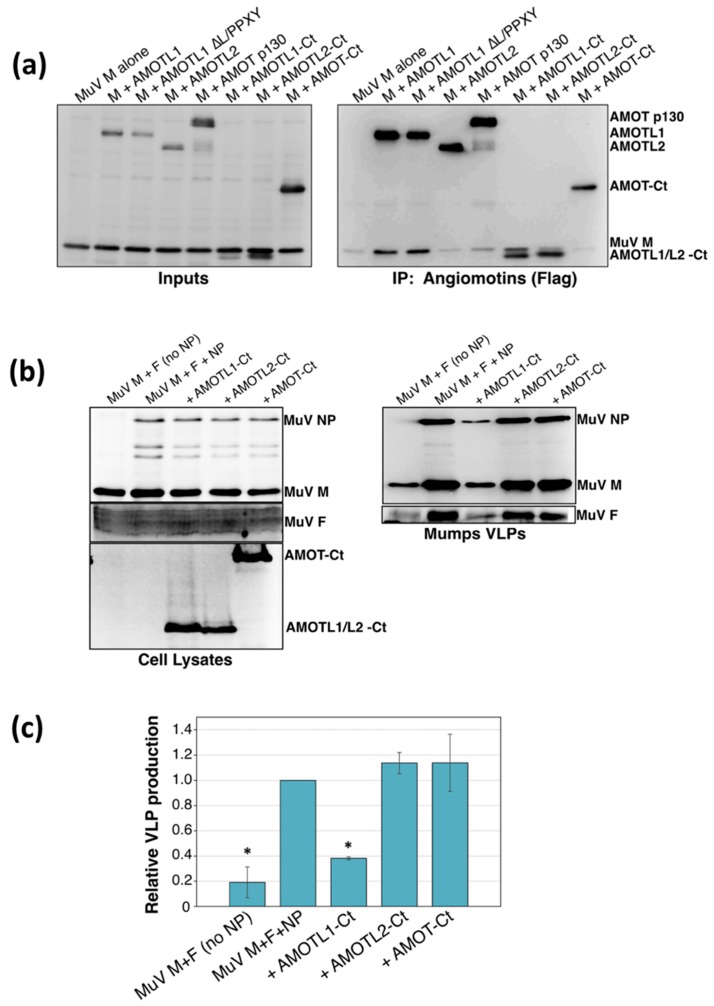
Mumps virus M protein binds to AMOTL1, but not to AMOT or AMOTL2. (**a**) 293T cells were transfected to produce mumps virus M protein together with the indicated Flag-tagged angiomotins. Cells were lysed in a solution containing 0.5% NP-40, and immunoprecipitation was carried out using a combination of anti-Flag and anti-mumps virus M antibodies (left), or using Flag antibody only (right). Proteins were fractionated on SDS gels and detected by immunoblotting. (**b**) 293T cells were transfected to produce mumps virus M, F, and NP proteins together with the indicated angiomotin-derived polypeptides. VLPs from culture supernatants were purified by centrifugation through sucrose cushions followed by flotation on sucrose gradients. Cell lysates and purified VLPs were fractionated on SDS gels and proteins were detected by immunoblotting. (**c**) Three independent experiments were performed as described for panel (**b**), and VLP production efficiencies were calculated as described in the legend to Figure 3. Error bars indicate standard deviations. Differences from the values obtained in the absence of polypeptide co-expression were assessed for statistical significance by using a two-tailed Student’s *t*-test, and *p* values less than 0.01 are denoted by an asterisk.

To test the applicability of these findings to other paramyxoviruses, additional binding and VLP production experiments were carried out using the mumps virus M protein. Similar to the PIV5 M protein, mumps virus M bound readily to AMOTL1 in transfected cells, but could not bind to AMOT or AMOTL2 (Figure 4a). Consistent with earlier studies on PIV5 M binding to AMOTL1 [24], mumps virus M binding to AMOTL1 occurred independently of the three AMOTL1 L/PPXY motifs (Figure 4a). AMOTL1-Ct bound to mumps virus M, but AMOT-Ct and AMOTL2-Ct did not (Figure 4a). Mumps VLP production was significantly impaired upon expression of AMOTL1-Ct, whereas AMOT-Ct and AMOTL2-Ct expression had almost no effect on mumps VLP production (Figure 4b). Collectively, these results indicate that paramyxovirus M protein binding is highly selective for AMOTL1, suggesting that the binding likely occurs in a way that is different from that directed by the more promiscuous HIV-1 Gag protein.

### 3.2. AMOTL1 Binds to Multiple NEDD4 Family Proteins via L/PPXY Motifs

**Figure 5:**
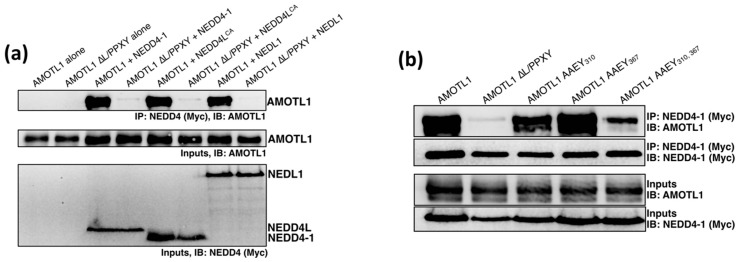
AMOTL1 binds to at least three NEDD4 family members through L/PPXY sequences. (**a**) 293T cells were transfected to produce AMOTL1 or AMOTL1 ∆L/PPXY together with Myc-tagged NEDD4-1, NEDD4L^CA^, or NEDL1. Cells were lysed in a solution containing 0.5% NP-40, and immunoprecipitation was carried out using Myc antibody. Proteins were fractionated on SDS gels and detected by immunoblotting using anti-AMOTL1 or anti-Myc antibodies, as indicated. (**b**) 293T cells were transfected to produce Myc-tagged NEDD4-1 together with the indicated AMOTL1 mutants having altered PPEY and/or LPTY sequences. Cells were lysed and co-immunoprecipitation analysis was carried out as described for panel (**a**).

AMOTL1 harbors two classical PPXY motifs together with one LPTY sequence, and these could, in theory, direct recruitment of the same WW domain-containing NEDD4 ubiquitin ligases that are known to facilitate the budding of many enveloped viruses. Two NEDD4 family members, NEDD4L (also known as NEDD4-2) [32,40] and NEDL2 (also known as HECW2) [41]) have already been shown to interact with AMOTL1. Here, we confirmed the interaction between AMOTL1 and NEDD4L, and also tested two additional NEDD4 family members for AMOTL1 binding, NEDD4-1 (also known as NEDD4) and NEDL1 (also known as HECW1). 293T cells were transfected to produce full-length AMOTL1 together with myc-tagged NEDD4 proteins (NEDD4L, NEDD4-1, or NEDL1). In preliminary experiments, NEDD4L expression was found to have a substantial negative effect on AMOTL1 stability, so these experiments employed a catalytically inactive NEDD4L variant (NEDD4L C942A, indicated henceforth as NEDD4L^CA^), allowing AMOTL1 stability to be maintained. NEDD4 proteins were immunoprecipitated via the myc tags, and co-precipitation of AMOTL1 was evaluated (Figure 5a). AMOTL1 was strongly co-immunoprecipitated in all three cases, indicating that this protein is capable of binding to multiple NEDD4 family members. To confirm that the binding in each case is dependent on L/PPXY sequences, we constructed AMOTL1 ∆L/PPXY in which the LPTY sequence is changed to AATY and the two PPEY sequences are each changed to AAEY. These mutations completely eliminated the ability of AMOTL1 to interact with any of three NEDD4 family proteins that were tested (Figure 5a). Hence, AMOTL1 binds to multiple NEDD4 family members, and binding occurs through L/PPXY motifs.

To examine the relative importance of the three L/PPXY motifs for NEDD4 interaction, and in particular to evaluate the importance of the nonclassical LPTY motif, we constructed an additional variant, AMOTL1 AAEY_310,367_ in which the two PPEY sequences at amino acid positions 310 and 367 have both been changed to AAEY, but the LPTY sequence at position 188 remains intact. This AMOTL1 mutant was expressed together with NEDD4-1 protein, and binding was evaluated by co-immunoprecipitation (Figure 5b). Although reduced compared to that observed with WT AMOTL1, significant binding to NEDD4-1 was still observed for AMOTL1 AAEY_310,367_, and this binding was almost completely eliminated in the case of AMOTL1 ∆L/PPXY where AMOTL1 is further modified to disrupt LPTY. This indicates that the LPTY sequence can function at least partially to mediate interaction with a NEDD4 family protein even in the absence of the two classical PPEY sequences. Of the two PPEY sequences, the one at position 310 likely plays a greater role in NEDD4-1 binding than the one at position 367, as disruption of PPEY_367_ alone had almost no effect on NEDD4-1 binding, while disruption of PPEY_310_ alone had a moderate impact on NEDD4-1 binding.

### 3.3. AMOTL1 Links NEDD4 Family Proteins to PIV5 M

**Figure 6:**
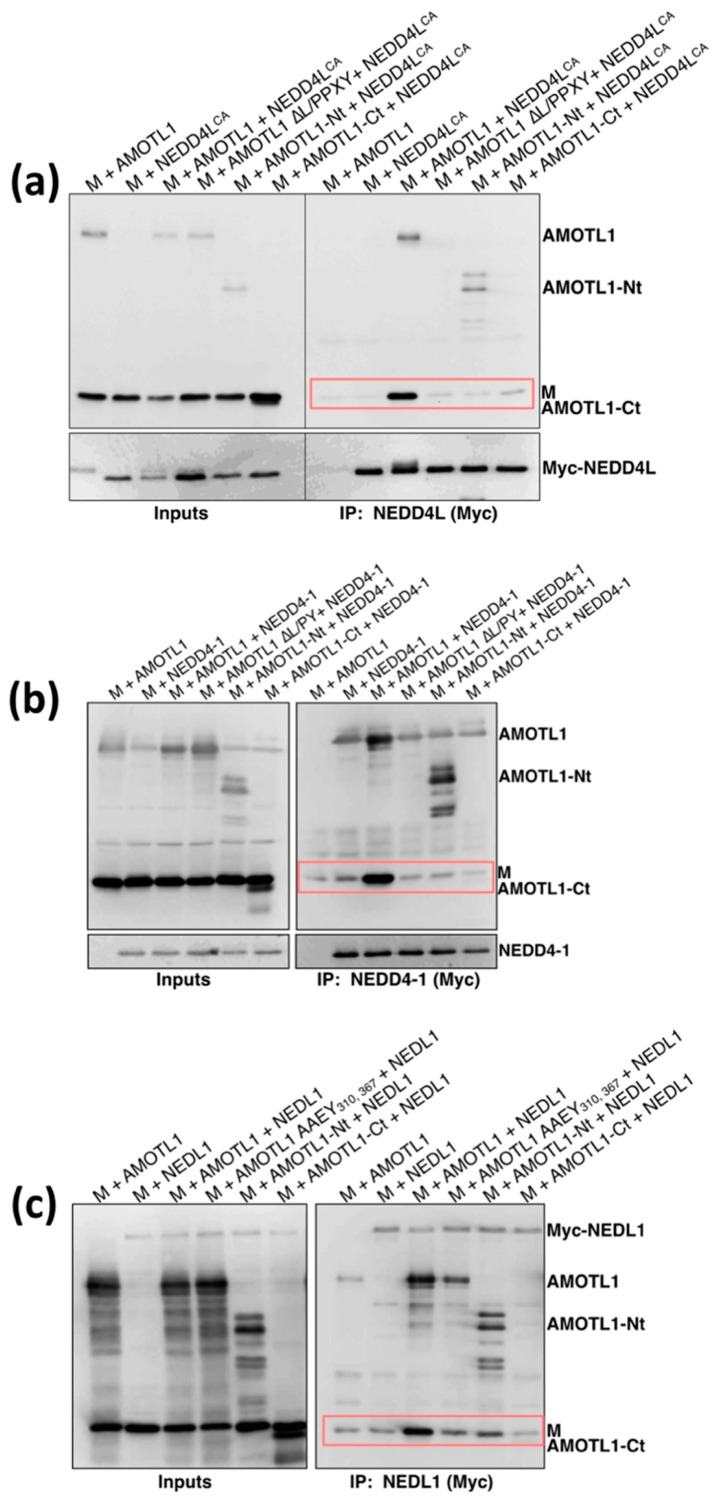
AMOTL1 links NEDD4 family proteins to PIV5 M. (**a**) 293T cells were transfected to produce AMOTL1 or the indicated AMOTL1 variants together with both PIV5 M protein and Myc-tagged NEDD4L^CA^. Cells were lysed in a solution containing 0.5% NP-40, and immunoprecipitation was carried out using Myc antibody. Proteins were fractionated on SDS gels and detected by immunoblotting using anti-PIV5 M, anti-AMOTL1, or anti-Myc antibodies, as indicated. Boxed regions denote M co-precipitation. (**b**) Three-way co-immunoprecipitation performed as in panel (**a**), but with Myc-tagged NEDD4-1 in place of NEDD4L^CA^. (**c**) Three-way co-immunoprecipitation performed as in panel (**a**), but with Myc-tagged NEDL1 in place of NEDD4L^CA^.

We next tested if PIV5 M can indirectly recruit NEDD4 family proteins through AMOTL1. PIV5 M, Flag-tagged AMOTL1, and Myc-tagged NEDD4L were expressed together in 293T cells (Figure 6a). Nedd4L was immunoprecipitated using Myc antibody and co-precipitation of AMOTL1 and M was evaluated. In the absence of AMOTL1 expression, M protein did not bind efficiently to NEDD4L. M co-immunoprecipitation in this case was similar to background levels (Figure 6a, lane 2). However, when AMOTL1 was expressed as a bridging protein, NEDD4L pulldown led to strong M co-immunoprecipitation (Figure 6a, lane 3). AMOTL1-Nt (illustrated in Figure 1) could not substitute for AMOTL1 as the bridging protein, because, although it binds to NEDD4L through its L/PPXY motifs, it lacks the ability to bind with PIV5 M [24]. Indeed, NEDD4L pulldown in this case led to efficient co-precipitation of AMOTL1-Nt, but poor co-precipitation of PIV5 M (Figure 6a, lane 5). Conversely, AMOTL1-Ct and AMOTL1 ∆L/PPXY are both able to bind to M, but cannot bind to NEDD4L due to lack of L/PPXY. Here, NEDD4L pulldown led to neither AMOTL1 nor M protein co-precipitation (Figure 6a, lanes 4 and 6). Hence, co-immunoprecipitation of M protein with NEDD4L occurred only in the presence of full-length AMOTL1 that contains L/PPXY sequences and has the ability to bind with PIV5 M.

Similar results were obtained when using NEDD4-1 (Figure 6b) or NEDL1 (Figure 6c) in place of NEDD4L. Only AMOTL1 that possesses L/PPXY motifs and the C-terminal M-binding region was able to act as a linker to allow M co-precipitation with NEDD4-1 or NEDL1. These results demonstrate that at least three different NEDD4 family proteins can be indirectly recruited to a paramyxovirus M protein through AMOTL1.

### 3.4. PIV5 M-PPXY Binds to NEDD4L without the Need for AMOTL1

**Figure 7:**
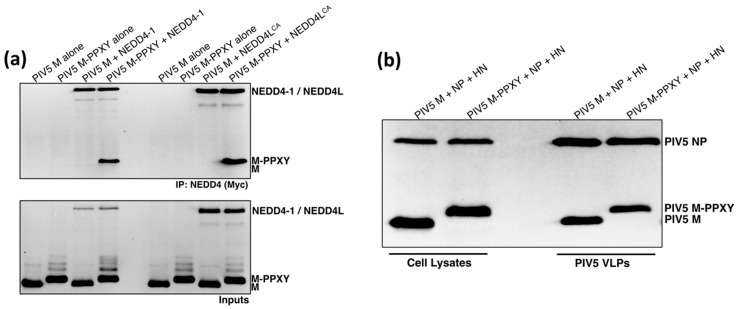
PIV5 M-PPXY binds to NEDD4-1 and NEDD4L without the need for AMOTL1. (**a**) 293T cells were transfected to produce PIV5 M or PIV5 M-PPXY that has the Rous sarcoma virus PPXY late domain attached to its C-terminal end, together with NEDD4-1 or NEDD4L^CA^. Cells were lysed in a solution containing 0.5% NP-40, and immunoprecipitation was carried out using Myc antibody. Proteins were fractionated on SDS gels and detected by immunoblotting using anti-PIV5 M and anti-Myc antibodies. (**b**) 293T cells were transfected to produce PIV5 M or PIV5 M-PPXY, together with PIV5 HN and NP proteins for VLP production. VLPs from culture supernatants were purified by centrifugation through sucrose cushions followed by flotation on sucrose gradients. Cell lysates and purified VLPs were fractionated on SDS gels and proteins were detected by immunoblotting.

Angiomotin-dependent recruitment of NEDD4 family proteins by paramyxoviruses and HIV-1 may represent an alternative to the more common strategy employed by other enveloped viruses in which PPXY late domains provide direct, angiomotin-independent NEDD4 binding. In theory, if paramyxovirus M proteins had PPXY late domains, they would no longer need AMOTL1 for NEDD4 recruitment. To test this idea, we appended a PPXY late domain derived from Rous sarcoma virus (RSV) Gag protein to the C-terminal end of PIV5 M protein. The modified M protein was expressed together with Myc-tagged NEDD4-1 or NEDD4L, and binding was assessed by co-immunoprecipitation. As expected, unmodified M protein had no appreciable ability to bind NEDD4-1 or NEDD4L (Figure 7a). However, M with the RSV late domain appended to it gained the ability to efficiently bind to both NEDD4-1 and NEDD4L. VLP production experiments were carried out to determine if the RSV late domain appendage affected the budding function of M protein. VLP budding directed by M-PPXY had similar efficiency to that directed by unmodified M protein (Figure 7b). Hence, there are seemingly no structural or functional barriers preventing the direct use of PPXY late domains within paramyxovirus M proteins for NEDD4 recruitment, yet these viruses have avoided the use of PPXY late domains and appear to recruit indirectly via AMOTL1 nonetheless.

## 4. Discussion

Many enveloped viruses employ PPXY sequences and other late domains to mediate recruitment of host ESCRT machinery for budding [4,6,42,43]. Details of how PPXY-type late domains connect ESCRT machinery to viral components at budding sites are not completely understood. However, recruitment of WW domain-containing NEDD4 family ubiquitin ligases and subsequent conjugation of ubiquitin to viral and/or cellular components does appear to be important for PPXY-dependent virus budding [4,6,7,44]. Several ESCRT proteins including ALIX and TSG101 contain ubiquitin-binding domains, and it has been proposed that ubiquitination of viral proteins thereby enhances ESCRT recruitment [44,45]. In many cases, PPXY late domains are not present in isolation, but instead appear alongside of P(T/S)AP or YPXL sequences, potentially allowing ESCRT engagement and virus budding to be facilitated in a cooperative manner [4]. Paramyxovirus M proteins generally lack PPXY and other classical late domain sequences, yet in many cases the budding of these viruses is ESCRT-dependent [3]. Here, we provide evidence that three NEDD4 family members (NEDD4-1, NEDD4L, and NEDL1) can be indirectly recruited to a paramyxovirus M protein via the PPXY-bearing host protein AMOTL1. The possibility that other NEDD4 family members such as ITCH, WWP1, and WWP2 could also be linked to M through AMOTL1 remains to be tested. Our findings support a new model for the budding of paramyxoviruses that explains the absence of PPXY late domains, as M protein binding to AMOTL1 would lead to indirect recruitment of the same NEDD4 ubiquitin ligases that are directly recruited by other viruses through PPXY late domains.

The importance of M protein ubiquitination for budding has been well established for many paramyxoviruses. For example, the PIV5 M protein is a target for ubiquitin attachment and mass spectrometry has identified specific lysine residues that are the preferred sites for ubiquitination [46]. Ubiquitination could be prevented through proteasome inhibitor treatment or removal of lysines, and in both cases M budding function was impaired, with M failing to concentrate normally at budding sites [46]. Several paramyxovirus M proteins undergo nuclear-cytoplasmic shuttling during the early phases of virus infection, and these M proteins become ubiquitinated at their nuclear localization sequences [47,48]. Inhibition of Nipah virus, Hendra virus, Sendai virus, or mumps virus M ubiquitination leads to retention of these proteins in the nucleolus, to the detriment of budding function [47,48]. Our results here suggest a potential mechanism for recruitment of NEDD4 family ubiquitin ligases to M proteins via AMOTL1. Further studies are needed to determine the importance of AMOTL1-mediated NEDD4 recruitment for proper ubiquitination and function of paramyxovirus M proteins.

The three angiomotin proteins have overlapping functions related to endothelial cell migration and control of YAP signaling [25], and all three are capable of binding to HIV-1 Gag protein [32]. This raised the possibility that there could also be a degree of redundancy among the angiomotins with respect to paramyxovirus budding function. However, we found that PIV5 and mumps virus M proteins bound only to AMOTL1, and not to AMOT or AMOTL2. Hence, the angiomotin binding activities of paramyxovirus M proteins seems to be different and more selective than that exhibited by HIV-1 Gag. It is unclear at present how the profile of NEDD4 family member recruitment would differ for a virus that uses AMOTL1 exclusively as the linker, as compared to one that uses a combination of angiomotins, and this will be an important avenue for further investigation.

Although NEDD4 recruitment is the most straightforward way to explain the importance of angiomotins for virus budding, there are compelling reasons to suspect that angiomotins provide added value to enveloped viruses during particle release beyond NEDD4 recruitment. First, angiomotins possess several features (F-actin binding [49], apical membrane distribution in polarized epithelial cells [29], and the presence of consensus membrane-bending BAR domain sequences [25,30]) that could provide benefits for virus budding irrespective of NEDD4 recruitment. Second, there are seemingly no structural or functional barriers preventing the direct use of PPXY late domains within the paramyxovirus M proteins (as evidenced by the NEDD4-binding and VLP production abilities of M-PPXY), yet these viruses recruit indirectly via AMOTL1 nonetheless. Finally, there are now two known examples of indirect NEDD4 recruitment for budding by evolutionarily distinct virus families (PIV5/mumps virus and HIV-1), and in both cases the intermediary protein is an angiomotin. This is despite the fact that a multitude of PPXY-containing, NEDD4-interacting proteins exists in cells that could in principle be used to indirectly link NEDD4 proteins to the viral matrix or Gag proteins. Defining specific NEDD4-dependent vs. NEDD4-independent contributions of AMOTL1 to paramyxovirus budding will be an important goal of future studies, and these should be facilitated by availability of M-PPXY proteins capable of recruiting NEDD4 proteins without the need for AMOTL1 linkers. Overall, our findings are consistent with a scenario in which paramyxovirus particle formation involves both NEDD4-dependent and NEDD4-independent functions for AMOTL1 which together lead to viral protein accumulation at budding sites, induction of membrane curvature, ubiquitination of viral and/or cellular components, recruitment of ESCRT machinery, and particle release through membrane fission.
